# Racial and Ethnic Disparities in COVID-19 Incidence by Age, Sex, and Period Among Persons Aged <25 Years — 16 U.S. Jurisdictions, January 1–December 31, 2020

**DOI:** 10.15585/mmwr.mm7011e1

**Published:** 2021-03-19

**Authors:** Miriam E. Van Dyke, Maria C.B. Mendoza, Wen Li, Erin M. Parker, Brook Belay, Elizabeth M. Davis, Joshua J. Quint, Ana Penman-Aguilar, Kristie E.N. Clarke

**Affiliations:** ^1^Epidemic Intelligence Service, CDC; ^2^CDC COVID-19 Response Team; ^3^New Mexico Department of Health; ^4^Hawaii State Department of Health; ^5^Office of Minority Health and Health Equity, CDC.

The COVID-19 pandemic has disproportionately affected racial and ethnic minority groups in the United States. Whereas racial and ethnic disparities in severe COVID-19–associated outcomes, including mortality, have been documented (*1*–*3*), less is known about population-based disparities in infection with SARS-CoV-2, the virus that causes COVID-19. In addition, although persons aged <30 years account for approximately one third of reported infections,^§^ there is limited information on racial and ethnic disparities in infection among young persons over time and by sex and age. Based on 689,672 U.S. COVID-19 cases reported to CDC’s case-based surveillance system by jurisdictional health departments, racial and ethnic disparities in COVID-19 incidence among persons aged <25 years in 16 U.S. jurisdictions^¶^ were described by age group and sex and across three periods during January 1–December 31, 2020. During January–April, COVID-19 incidence was substantially higher among most racial and ethnic minority groups compared with that among non-Hispanic White (White) persons (rate ratio [RR] range = 1.09–4.62). During May–August, the RR increased from 2.49 to 4.57 among non-Hispanic Native Hawaiian and Pacific Islander (NH/PI) persons but decreased among other racial and ethnic minority groups (RR range = 0.52–2.82). Decreases in disparities were observed during September–December (RR range = 0.37–1.69); these decreases were largely because of a greater increase in incidence among White persons, rather than a decline in incidence among racial and ethnic minority groups. NH/PI, non-Hispanic American Indian or Alaska Native (AI/AN), and Hispanic or Latino (Hispanic) persons experienced the largest persistent disparities over the entire period. Ensuring equitable and timely access to preventive measures, including testing, safe work and education settings, and vaccination when eligible is important to address racial/ethnic disparities.

Population-based COVID-19 incidence (cases per 100,000 persons) by race and ethnicity, sex, and age was calculated for January 1–December 31, 2020, overall, and for three approximately equal 4-month periods (January 1–April 30, May 1–August 31, and September 1–December 31) using COVID-19 cases reported to CDC’s case-based surveillance system** by jurisdictional health departments. Incompleteness of race and ethnicity data is a widespread challenge in analyses of COVID-19 disparities.^††^ To minimize the impact of missing data, jurisdictions selected for analyses reported ≥30% of the total number of jurisdictional aggregate cases^§§^ to CDC and had ≥70% of race and ethnicity information complete among cases reported during January 1–December 31, 2020. Fifteen U.S. states and the District of Columbia were included, with a total of 689,672 cases among persons aged <25 years with information on race and ethnicity and sex.^¶¶^ Population denominators were obtained from the 2019 U.S. Census Bureau’s Annual County Resident Population Estimates by Age, Sex, Race, and Hispanic Origin.***

Seven racial and ethnic categories (AI/AN, non-Hispanic Asian [Asian], non-Hispanic Black or African-American [Black], NH/PI, White, Hispanic, and non-Hispanic multiple race [multiracial]) and five age categories (0–4, 5–9, 10–14, 15–19, and 20–24 years) were examined. RRs with 95% confidence intervals (CIs) comparing rates by race and ethnicity (combined), age, and/or sex overall and for each period were calculated. Statistical analyses were conducted using SAS software (version 9.4; SAS Institute). Rate ratios with 95% CIs excluding 1.0 were considered to be statistically significant. This activity was reviewed by CDC and was conducted consistent with applicable federal law and CDC policy.^†††^

The sample of 689,672 cases included 15,068 (2%) cases identified during January–April; 177,778 (26%) during May–August and 496,826 (72%) during September–December (Table 1). During January–April, COVID-19 incidence ranged from 35 cases per 100,000 among White persons to 163 per 100,000 among AI/AN persons. Compared with White persons, rates were higher among AI/AN (RR = 4.62), Hispanic (RR = 3.87), NH/PI (RR = 2.49), Black (RR = 2.46), and Asian persons (RR = 1.53) and were approximately equal among multiracial persons (RR = 1.09).

**TABLE 1:** COVID–19 incidence* and rate ratios, by race/ethnicity, sex, and age group among persons aged <25 years across three periods — 16 U.S. jurisdictions,^†^ January 1–December 31, 2020

Date/Characteristic	No. of cases	Cases per 100,000 population (95% CI)	RR (95% CI)
**January 1–April 30, 2020**			
**All**	**15,068**	**63 (62–64)**	**—**
**Sex**			
Male	6,884	57 (55–58)	0.80 (0.78–0.83)
Female	8,184	70 (69–72)	Ref
**Race/Ethnicity**			
AI/AN, non-Hispanic	536	163 (150–177)	4.62 (4.22–5.05)
Asian, non-Hispanic	498	54 (49–59)	1.53 (1.39–1.67)
Black, non-Hispanic	2,461	87 (83–90)	2.46 (2.34–2.58)
NH/PI, non-Hispanic	73	88 (70–111)	2.49 (1.98–3.14)
White, non-Hispanic	4,947	35 (34–36)	Ref
Hispanic/Latino	6,129	137 (133–140)	3.87 (3.73–4.02)
Multiple, non-Hispanic	424	38 (35–42)	1.09 (0.98–1.20)
**Age group (yrs)**			
0–4	956	21 (20–23)	1.28 (1.17–1.41)
5–9	772	17 (16–18)	Ref
10–14	1,184	25 (23–26)	1.49 (1.36–1.63)
15–19	3,267	67 (65–70)	4.03 (3.72–4.36)
20–24	8,889	175 (171–178)	10.47 (9.72–11.26)
**May 1–August 31, 2020**			
**All**	**177,778**	**747 (744–751)**	**—**
**Sex**			
Male	84,270	693 (688–698)	0.86 (0.85–0.87)
Female	93,508	804 (799–809)	Ref
**Race/Ethnicity**			
AI/AN, non-Hispanic	3,245	986 (952–1,020)	1.86 (1.80–1.93)
Asian, non-Hispanic	3,781	409 (396–422)	0.77 (0.75–0.80)
Black, non-Hispanic	24,501	862 (852–873)	1.63 (1.61–1.65)
NH/PI, non-Hispanic	2,007	2,418 (2,314–2,526)	4.57 (4.37–4.77)
White, non-Hispanic	74,259	530 (526–533)	Ref
Hispanic/Latino	66,938	1,493 (1,481–1,504)	2.82 (2.79–2.85)
Multiple, non-Hispanic	3,047	275 (266–285)	0.52 (0.50–0.54)
**Age group (yrs)**			
0–4	14,017	314 (309–319)	1.01 (0.98–1.03)
5–9	14,406	312 (307–317)	Ref
10–14	20,490	430 (424–436)	1.38 (1.35–1.41)
15–19	50,210	1,034 (1,025–1,043)	3.32 (3.26–3.38)
20–24	78,655	1,547 (1,536–1,557)	4.96 (4.88–5.05)
**September 1–December 31, 2020**			
**All**	**496,826**	**2,088 (2,082–2,094)**	**—**
**Sex**			
Male	236,237	1,943 (1,935–1,951)	0.87 (0.86–0.87)
Female	260,589	2,240 (2,231–2,248)	Ref
**Race/Ethnicity**			
AI/AN, non-Hispanic	11,870	3,605 (3,541–3,671)	1.62 (1.59–1.65)
Asian, non-Hispanic	11,680	1,263 (1,240–1,286)	0.57 (0.56–0.58)
Black, non-Hispanic	32,200	1,133 (1,121–1,146)	0.51 (0.50–0.52)
NH/PI, non-Hispanic	3,119	3,757 (3,628–3,891)	1.69 (1.63–1.75)
White, non-Hispanic	311,591	2,222 (2,214–2,230)	Ref
Hispanic/Latino	117,305	2,616 (2,601–2,631)	1.18 (1.17–1.19)
Multiple, non-Hispanic	9,061	819 (803–836)	0.37 (0.36–0.38)
**Age group (yrs)**			
0–4	33,595	752 (744–760)	0.71 (0.70–0.72)
5–9	48,824	1,056 (1,047–1,066)	Ref
10–14	76,922	1,615 (1,604–1,627)	1.53 (1.51–1.55)
15–19	149,660	3,083 (3,067–3,098)	2.92 (2.89–2.95)
20–24	187,825	3,693 (3,677–3,710)	3.50 (3.46–3.53)
**January 1–December 31, 2020**			
**All**	**689,672**	**2,899 (2,892–2,906)**	**—**
**Sex**			
Male	327,391	2,693 (2,684–2,702)	0.86 (0.86–0.87)
Female	362,281	3,114 (3,104–3,124)	Ref
**Race/Ethnicity**			
AI/AN, non-Hispanic	15,651	4,754 (4,680–4,829)	1.71 (1.68–1.73)
Asian, non-Hispanic	15,959	1,725 (1,699–1,752)	0.62 (0.61–0.63)
Black, non-Hispanic	59,162	2,083 (2,066–2,099)	0.75 (0.74–0.75)
NH/PI, non-Hispanic	5,199	6,263 (6,095–6,436)	2.25 (2.19–2.31)
White, non-Hispanic	390,797	2,787 (2,778–2,795)	Ref
Hispanic/Latino	190,372	4,245 (4,226–4,264)	1.52 (1.52–1.53)
Multiple, non-Hispanic	12,532	1,133 (1,113–1,153)	0.41 (0.40–0.41)
**Age group (yrs)**			
0–4	48,568	1,087 (1,078–1,097)	0.79 (0.78–0.79)
5–9	64,002	1,385 (1,374–1,395)	Ref
10–14	98,596	2,070 (2,057–2,083)	1.50 (1.48–1.51)
15–19	203,137	4,184 (4,166–4,202)	3.02 (2.99–3.05)
20–24	275,369	5,415 (5,394–5,435)	3.91 (3.88–3.94)

**Abbreviations:** AI/AN = American Indian or Alaska Native; CI = confidence interval; NH/PI = Native Hawaiian and Pacific Islander; Ref = referent group; RR = rate ratio.

* Rates for each period and for the full period were calculated using the following equation: (cases/population) x 100,000 persons. COVID–19 cases were identified using CDC’s Data Collation and Integration for Public Health Event Response system (https://data.cdc.gov/browse?tags=covid-19 [accessed January 27, 2021]). Case surveillance data were received directly from two jurisdictional health departments (Hawaii State Department of Health and New Mexico Department of Health) for all racial/ethnic groups to allow for separate reporting of NH/PI persons. Population estimates were provided by the 2019 U.S. Census Bureau’s Annual County Resident Population Estimates by Age, Sex, Race, and Hispanic Origin (https://www.census.gov/programs-surveys/popest/technical-documentation/file-layouts.html [accessed August 20, 2020]). 2019 population estimates in the 16 selected jurisdictions were as follows: persons aged <25 years: all (23,792,864), males (12,157,933), females (11,634,931), non-Hispanic AI/AN (329,235), non-Hispanic Asian (925,072), non-Hispanic Black or African-American (2,840,777), non-Hispanic NH/PI (83,012), non-Hispanic White (14,024,304), Hispanic or Latino (4,484,434), and non-Hispanic persons of multiple races (1,106,030); and persons aged 0–4 years (4,467,369), 5–9 years (4,622,261), 10–14 years (4,762,433), 15–19 years (4,855,127), and 20–24 years (5,085,674). No measures were calculated for non-Hispanic persons of other races with COVID-19 (n = 19,627) because of lack of population denominator information from U.S. Census Bureau.

^†^ Arkansas, District of Columbia, Florida, Hawaii, Kansas, Kentucky, Maine, Massachusetts, Michigan, Minnesota, New Mexico, Oklahoma, Oregon, Utah, Vermont, and Wisconsin.

From January–April to May–August, COVID-19 incidence increased among all racial and ethnic groups, ranging from 275 per 100,000 among multiracial persons to 2,418 per 100,000 among NH/PI persons. The largest relative increase occurred among NH/PI persons, with incidence increasing 26-fold, from 88 to 2,418 per 100,000. Rate ratios increased among NH/PI persons but decreased among other racial and ethnic minority groups. During May–August, compared with that among White persons, incidence remained higher among NH/PI (RR = 4.57), Hispanic (RR = 2.82), AI/AN (RR = 1.86), and Black persons (RR = 1.63), but was lower among Asian (RR = 0.77) and multiracial persons (RR = 0.52).

From May–August to September–December, COVID-19 incidence increased among all racial and ethnic groups. The largest relative increase occurred among White persons, with incidence increasing approximately 320%, from 530 to 2,222 cases per 100,000 from May–August to September–December. Disparities decreased among all racial and ethnic minority groups. During September–December, compared with that among White persons, incidence remained higher among NH/PI (RR = 1.69), AI/AN (RR = 1.62), and Hispanic persons (RR = 1.18), but was lower among Asian (RR = 0.57), Black (RR = 0.51), and multiracial persons (RR = 0.37).

Incidence was higher among females than among males during all of 2020 and across periods. Incidence also tended to be lowest among younger children across periods. Lowest incidence was observed among children aged 5–9 years during January–April, those aged 0–9 years during May–August, and those aged 0–4 years during September–December.

During January–December, overall, the highest COVID-19 incidence relative to that among White persons was among NH/PI persons of most age groups, with the largest differences among those aged 0–4 (RR = 4.03) and 5–9 years (RR = 3.21) (Figure) (Supplementary Table, https://stacks.cdc.gov/view/cdc/103733). During January–December, among persons aged ≤14 years, incidence relative to White persons was initially higher among Black and Asian persons and persistently higher among NH/PI, AI/AN, and Hispanic persons; among persons aged 15–24 years, incidence relative to White persons was initially higher among Black, Asian, and multiracial persons, and persistently higher among NH/PI, AI/AN, and Hispanic persons. Overall, during January–December, differences compared with White persons among AI/AN, NH/PI, and Hispanic persons were larger in persons aged ≤14 years than among those aged 15–24 years. Racial and ethnic disparities were similar in magnitude and direction for both females and males across age groups (Table 2).

**FIGURE:**
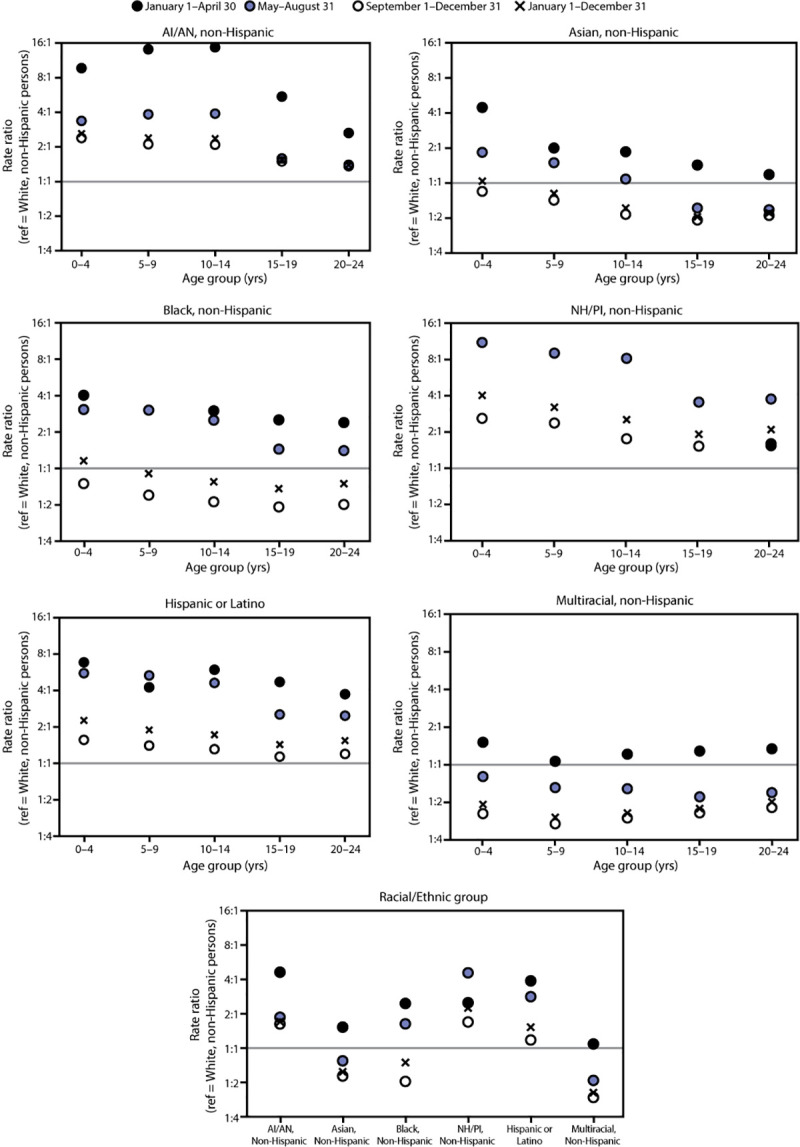
Rate ratios* comparing COVID-19 incidence^†^ among racial and ethnic minority persons to COVID-19 incidence among non-Hispanic White persons, among persons aged <25 years, by age group in three periods — 16 U.S. jurisdictions,^§^ January 1–December 31, 2020 **Abbreviations:** AI/AN=American Indian or Alaska Native; NH/PI=Native Hawaiian and Pacific Islander; ref = referent group. * Rate ratios were calculated during each period and overall. Data used to generate this figure are included in the Supplementary Table, https://stacks.cdc.gov/view/cdc/103733. Rate ratios are not available in situations where data were suppressed because of <20 cases being reported for a given race/ethnicity and age group during a period. During January 1–April 30, 2020, <20 cases were reported for non-Hispanic NH/PI persons aged 0–4, 5–9, 10–14, and 15–19 years. Rate ratios were similar and thus corresponding rate ratio symbols overlap in the figure for the following categories: AI/AN persons aged 15–19 and 20–24 years during May 1–August 31 and September 1–December 31; Black persons aged 5–9 years during January 1–April 30 and May 1–August 31; and NH/PI persons aged 20–24 years during January 1–April 30 and September 1–December 31. † Rates for each period and for the full period were calculated using the following equation: (cases/population) x 100,000 persons. COVID-19 cases were identified using CDC’s Data Collation and Integration for Public Health Event Response system (https://data.cdc.gov/browse?tags=covid-19 [accessed January 27, 2021]). Case surveillance data were received directly from two jurisdictional health departments (Hawaii State Department of Health and New Mexico Department of Health) for all racial/ethnic groups to allow for separate reporting of NH/PI persons. Population estimates were provided by the 2019 U.S. Census Bureau’s Annual County Resident Population Estimates by Age, Sex, Race, and Hispanic Origin (https://www.census.gov/programs-surveys/popest/technical-documentation/file-layouts.html [accessed August 20, 2020]). § Arkansas, District of Columbia, Florida, Hawaii, Kansas, Kentucky, Maine, Massachusetts, Michigan, Minnesota, New Mexico, Oklahoma, Oregon, Utah, Vermont, and Wisconsin.

**TABLE 2:** Sex-specific COVID-19 incidence* and rate ratios among persons aged <25 years, by age group, sex, and race/ethnicity — 16 U.S. jurisdictions,^†^ January 1–December 31, 2020

Age group, race/ethnicity	Sex					
	Female			Male		
	No. of cases	Cases per 100,000 population (95% CI)	Rate ratio (95% CI)	No. of cases	Cases per 100,000 population (95% CI)	Rate ratio (95% CI)
**0–4 yrs**						
**All**	**23,272**	**1,067 (1,053–1,081)**	**—**	**25,296**	**1,107 (1,093–1,120)**	**—**
AI/AN, non-Hispanic	658	2,247 (2,082–2,425)	2.69 (2.49–2.91)	677	2,208 (2,048–2,381)	2.54 (2.35–2.75)
Asian, non-Hispanic	642	858 (794–927)	1.03 (0.95–1.11)	721	913 (849–982)	1.05 (0.98–1.13)
Black, non-Hispanic	2,541	940 (904–977)	1.13 (1.08–1.18)	2,844	1,028 (991–1,067)	1.18 (1.14–1.23)
NH/PI, non-Hispanic	266	3,576 (3,171–4,033)	4.28 (3.79–4.83)	258	3,306 (2,926–3,735)	3.81 (3.37–4.31)
White, non-Hispanic	10,391	835 (820–852)	Ref	11,382	868 (852–884)	Ref
Hispanic/Latino	8,299	1,901 (1,861–1,943)	2.28 (2.21–2.34)	8,889	1,951 (1,910–1,992)	2.25 (2.19–2.31)
Multiple, non-Hispanic	475	399 (365–436)	0.48 (0.44–0.52)	525	420 (386–458)	0.48 (0.44–0.53)
**5–9 yrs**						
**All**	**31,333**	**1,389 (1,374–1,404)**	**—**	**32,669**	**1,381 (1,366–1,396)**	**—**
AI/AN, non-Hispanic	917	2,901 (2,719–3,095)	2.40 (2.24–2.56)	941	2,861 (2,684–3,050)	2.39 (2.24–2.56)
Asian, non-Hispanic	741	904 (841–971)	0.75 (0.69–0.80)	890	1,048 (981–1,119)	0.88 (0.82–0.94)
Black, non-Hispanic	3,019	1,081 (1,043–1,120)	0.89 (0.86–0.93)	3,155	1,096 (1,058–1,135)	0.92 (0.88–0.95)
NH/PI, non-Hispanic	287	3,676 (3,275–4,127)	3.04 (2.70–3.42)	326	4,040 (3,624–4,503)	3.38 (3.03–3.77)
White, non-Hispanic	15,609	1,210 (1,191–1,229)	Ref	16,280	1,195 (1,177–1,214)	Ref
Hispanic/Latino	10,174	2,271 (2,227–2,315)	1.88 (1.83–1.92)	10,564	2,264 (2,221–2,308)	1.89 (1.85–1.94)
Multiple, non-Hispanic	586	501 (462–543)	0.41 (0.38–0.45)	513	414 (380–452)	0.35 (0.32–0.38)
**10–14 yrs**						
**All**	**49,235**	**2,112 (2,094–2,131)**	**—**	**49,361**	**2,030 (2,012–2,048)**	**—**
AI/AN, non-Hispanic	1,477	4,461 (4,239–4,694)	2.31 (2.19–2.44)	1,520	4,492 (4,272–4,723)	2.42 (2.30–2.55)
Asian, non-Hispanic	949	1,089 (1,022–1,160)	0.56 (0.53–0.60)	1,066	1,210 (1,140–1,285)	0.65 (0.61–0.69)
Black, non-Hispanic	4,192	1,510 (1,465–1,556)	0.78 (0.76–0.81)	4,042	1,416 (1,373–1,461)	0.76 (0.74–0.79)
NH/PI, non-Hispanic	424	4,803 (4,367–5,283)	2.49 (2.26–2.74)	445	4,779 (4,355–5,245)	2.58 (2.35–2.83)
White, non-Hispanic	26,147	1,930 (1,907–1,954)	Ref	26,360	1,853 (1,831–1,876)	Ref
Hispanic/Latino	15,128	3,335 (3,282–3,389)	1.73 (1.69–1.76)	15,020	3,175 (3,125–3,226)	1.71 (1.68–1.75)
Multiple, non-Hispanic	918	794 (744–847)	0.41 (0.39–0.44)	908	760 (712–811)	0.41 (0.38–0.44)
**15–19 yrs**						
**All**	**109,350**	**4,601 (4,574–4,628)**	**—**	**93,787**	**3,784 (3,760–3,808)**	**—**
AI/AN, non-Hispanic	2,432	7,218 (6,937–7,511)	1.55 (1.49–1.61)	1,971	5,634 (5,391–5,889)	1.54 (1.47–1.61)
Asian, non-Hispanic	2,133	2,181 (2,090–2,275)	0.47 (0.45–0.49)	1,960	2,065 (1,975–2,158)	0.56 (0.54–0.59)
Black, non-Hispanic	8,056	2,915 (2,852–2,979)	0.63 (0.61–0.64)	7,774	2,715 (2,655–2,776)	0.74 (0.72–0.76)
NH/PI, non-Hispanic	715	8,679 (8,066–9,339)	1.86 (1.73–2.01)	633	7,253 (6,709–7,840)	1.98 (1.83–2.14)
White, non-Hispanic	66,431	4,655 (4,620–4,691)	Ref	54,869	3,661 (3,630–3,691)	Ref
Hispanic/Latino	27,571	6,361 (6,286–6,436)	1.37 (1.35–1.39)	24,846	5,497 (5,430–5,566)	1.50 (1.48–1.52)
Multiple, non-Hispanic	2,012	2,010 (1,924–2,100)	0.43 (0.41–0.45)	1,734	1,689 (1,612–1,771)	0.46 (0.44–0.48)
**20–24 yrs**						
**All**	**149,091**	**5,987 (5,957–6,018)**	**—**	**126,278**	**4,865 (4,839–4,892)**	**—**
AI/AN, non-Hispanic	2,881	8,386 (8,086–8,698)	1.43 (1.38–1.48)	2,177	6,253 (5,995–6,521)	1.34 (1.29–1.40)
Asian, non-Hispanic	3,558	2,997 (2,900–3,097)	0.51 (0.49–0.53)	3,299	2,805 (2,711–2,903)	0.60 (0.58–0.62)
Black, non-Hispanic	12,708	4,316 (4,241–4,391)	0.74 (0.72–0.75)	10,831	3,534 (3,468–3,601)	0.76 (0.74–0.78)
NH/PI, non-Hispanic	941	11,453 (10,744–12,209)	1.95 (1.83–2.08)	904	10,546 (9,880–11,256)	2.27 (2.12–2.42)
White, non-Hispanic	89,490	5,867 (5,829–5,906)	Ref	73,838	4,649 (4,615–4,682)	Ref
Hispanic/Latino	36,744	8,775 (8,685–8,865)	1.50 (1.48–1.51)	33,137	7,418 (7,339–7,499)	1.60 (1.58–1.62)
Multiple, non-Hispanic	2,769	3,059 (2,947–3,175)	0.52 (0.50–0.54)	2,092	2,251 (2,156 –2,349)	0.48 (0.46–0.51)

**Abbreviations:** AI/AN = American Indian or Alaska Native; CI = confidence interval; NH/PI = Native Hawaiian and Pacific Islander; Ref = referent group.

* Rates for each period and for the full period were calculated using the following equation: (cases/population) x 100,000 persons. COVID-19 cases were identified using CDC’s Data Collation and Integration for Public Health Event Response system (https://data.cdc.gov/browse?tags=covid-19 [accessed January 27, 2021]). Case surveillance data were received directly from two jurisdictional health departments (Hawaii State Department of Health and New Mexico Department of Health) for all racial/ethnic groups to allow for separate reporting of NH/PI persons. Population estimates were provided by the 2019 U.S. Census Bureau’s Annual County Resident Population Estimates by Age, Sex, Race, and Hispanic Origin (https://www.census.gov/programs-surveys/popest/technical-documentation/file-layouts.html [accessed August 20, 2020]).

^†^ Arkansas, District of Columbia, Florida, Hawaii, Kansas, Kentucky, Maine, Massachusetts, Michigan, Minnesota, New Mexico, Oklahoma, Oregon, Utah, Vermont, and Wisconsin.

## Discussion

Analysis of CDC’s case-based surveillance data in 16 U.S. jurisdictions during January–December 2020 indicates that racial and ethnic differences in COVID-19 incidence among persons aged <25 years changed over time. Disparities were substantial early in the pandemic among most racial and ethnic minority groups compared with White persons and then decreased over time, largely because of a greater increase in incidence among White persons. Among NH/PI persons, disparities increased from January–April to May–August and then decreased by September–December. The largest persistent disparities in COVID-19 incidence were among NH/PI, AI/AN, and Hispanic persons. Other studies have reported disproportionately higher percentages of COVID-19 cases among Hispanic, Black, Asian, and AI/AN children (*4*,*5*); however, no published studies to date have described national COVID-19 incidence among NH/PI children. 

Social determinants of health influence racial and ethnic disparities in case incidence.^§§§^ The large racial and ethnic COVID-19 disparities identified early in the pandemic in this analysis might reflect differential ability to participate in early mitigation measures, such as stay-at-home orders (*6*). Racial and ethnic minority groups are disproportionately represented in essential work settings, making it difficult for youths and parents to stay at home; a higher likelihood of living in a multigenerational household also increases the risk for household exposures to SARS-CoV-2.^¶¶¶^ For example, NH/PI persons, a group with some of the largest persistent disparities in this analysis, most often reside in multigenerational homes compared with other racial and ethnic groups (*7*). Despite on average having lower income and educational attainment, NH/PI persons are often grouped in analyses with Asian persons (*8*), thereby obscuring disparities influenced by these social determinants of health.

The decrease in racial and ethnic disparities observed over time was largely driven by a greater increase in COVID-19 incidence among White persons, rather than a decrease among racial and ethnic minority groups. This narrowing in differences should be considered in the context of geographic aspects of community spread over time and potential changes in access to or participation in mitigation measures or testing over time by race and ethnicity. For example, future studies could consider whether variations in state-mandated mitigation policies and other aspects of the policy environment led to the observed differential adherence in some mitigation measures by race/ethnicity (*9*). Further study of whether some testing strategies (e.g., repeat testing of students in some academic settings****) might have been differentially available by race and ethnicity over time is also needed.

The findings in this report are subject to at least five limitations. First, reporting of detailed case data and race and ethnicity to CDC is incomplete. Although this analysis was restricted to 16 jurisdictions with more complete case and race and ethnicity information, 23% of cases from these jurisdictions were missing data on race and ethnicity. Differences in data completeness by race and ethnicity could lead to underestimation of disparities (*10*). Restriction to 16 jurisdictions also limits the generalizability of these findings, because they are based on only 23% of the national population of persons aged <25 years; in addition, disparities could vary at geographic subdivisions within states. Second, these data likely underestimate the incidence of COVID-19 among persons aged <25 years because individual-level cases reported to CDC represent a portion of jurisdictional aggregate cases and asymptomatic persons are less likely to be tested. Third, cases among racial and ethnic minority groups might be disproportionately underreported given disparities in access to testing, leading to underestimation of disparities. Fourth, potential differences in testing, reporting, and completeness of data by race and ethnicity over time call for caution in interpretation of the observed changes in racial and ethnic disparities in this report. Finally, racial and ethnic disparities in COVID-19 incidence (and changes over time) might not reflect disparities in severe outcomes (*1*–*3*).^††††^

During January 1–December 31, 2020, substantial racial and ethnic disparities in COVID-19 incidence, observed early in the pandemic among persons aged <25 years in 16 jurisdictions, decreased over time, driven largely by a greater increase in reporting of cases among White persons. The largest persistent disparities were among NH/PI, AI/AN, and Hispanic persons. Ensuring equitable and timely access to preventive measures, including testing, safe work and education settings and vaccination when eligible is important to address racial/ethnic disparities.^§§§§^

**Summary**

**What is already known about this topic?**

U.S. racial and ethnic minority groups have been disproportionately affected by COVID-19.

**What is added by this report?**

Racial and ethnic disparities in COVID-19 incidence among persons aged <25 years in 16 U.S. jurisdictions evolved during the pandemic. Disparities were substantial during January–April and generally decreased during May–December, largely because of a greater increase in incidence among White persons, rather than a decline among racial and ethnic minority groups. The largest persistent disparities involved Native Hawaiian and Pacific Islander, American Indian or Alaska Native, and Hispanic persons.

**What are the implications for public health practice?**

Ensuring equitable and timely access to preventive measures, including testing, safe work and education settings, and vaccination when eligible is important to address racial/ethnic disparities.

## Footnotes

* These authors contributed equally as first authors.

† These authors contributed equally as senior authors.

§ https://covid.cdc.gov/covid-data-tracker/ (accessed February 14, 2021)

¶ Arkansas, District of Columbia, Florida, Hawaii, Kansas, Kentucky, Maine, Massachusetts, Michigan, Minnesota, New Mexico, Oklahoma, Oregon, Utah, Vermont, and Wisconsin.

** CDC implemented a data integration and management platform, Data Collation and Integration for Public Health Event Response (DCIPHER), for use in outbreak responses (https://data.cdc.gov/browse?tags = covid-19). This platform enables jurisdictions to directly enter or import and view their data. Individual-level case report data for COVID-19 cases were accessed through the DCIPHER system on January 27, 2021. Data were classified using the earliest available date related to the illness, specimen collection or reporting to CDC. Case surveillance data were received directly from two jurisdictional health departments (Hawaii State Department of Health and New Mexico Department of Health) for all racial/ethnic groups to allow for separate reporting of NH/PI persons. Data from these two jurisdictions were combined in analyses with data accessed through the DCIPHER system from the 14 other jurisdictions.

††
https://www.cdc.gov/coronavirus/2019-ncov/covid-data/faq-surveillance.html

§§ Aggregate counts from reporting jurisdictions were downloaded through HHS Protect Public Database. https://protect-public.hhs.gov/ (accessed January 27, 2021)

¶¶ Among the identified 919,652 persons aged <25 years with COVID-19 in the 16 jurisdictions, 210,353 (23%) persons were missing information on race and ethnicity and/or sex during January 1–December 31, 2020. Among the 210,353 persons missing information on race and ethnicity and/or sex, 207,659 (99%) were missing information on race and ethnicity. The percentages of persons aged <25 years with COVID-19 in the 16 jurisdictions missing information on race and ethnicity and/or sex were 20.2% during January 1–April 30, 18.9% during May 1–August 31, and 24.3% during September 1–December 31.

*** https://www.census.gov/programs-surveys/popest/technical-documentation/file-layouts.html (accessed August 20, 2020)

††† 45 C.F.R. part 46.102(l)(2), 21 C.F.R. part 56; 42 U.S.C. Sect. 241(d); 5 U.S.C. Sect. 552a; 44 U.S.C. Sect. 3501 et seq.

§§§
https://www.cdc.gov/coronavirus/2019-ncov/community/health-equity/race-ethnicity.html

¶¶¶
https://www.cdc.gov/coronavirus/2019-ncov/community/health-equity/racial-ethnic-disparities/increased-risk-exposure.html

****
https://www.washingtonpost.com/local/education/welcome-to-college-now-get-tested-for-the-coronavirus--again-and-again/2020/09/04/2d087722-ed2f-11ea-b4bc-3a2098fc73d4_story.html

††††
https://www.cdc.gov/coronavirus/2019-ncov/community/health-equity/racial-ethnic-disparities/disparities-illness.html

§§§§
https://www.cdc.gov/coronavirus/2019-ncov/community/health-equity/racial-ethnic-disparities/what-we-do.html
